# Reducing repeat pregnancies in adolescence: applying realist principles as part of a mixed-methods systematic review to explore what works, for whom, how and under what circumstances

**DOI:** 10.1186/s12884-016-1066-x

**Published:** 2016-09-20

**Authors:** Joanna M. Charles, Jo Rycroft-Malone, Rabeea’h Aslam, Maggie Hendry, Diana Pasterfield, Rhiannon Whitaker

**Affiliations:** 1 Centre for Health Economics & Medicines Evaluation, Ardudwy, Normal Site, Bangor University, Bangor, Gwynedd LL57 2PZ UK; 2 School of Healthcare Sciences, Fron Heulog, Bangor University, Bangor, Gwynedd LL57 2EF UK; 3 Liverpool Review and Implementation Group, University of Liverpool, Whelan Building, Brownlow Hill, Liverpool, L69 3GB UK; 4 North Wales Centre for Primary Care Research, Gwenfro Units 4-8, Wrexham Technology Park, Wrexham, UK; 5 Whitaker Research Ltd. Cae Ffos, Treborth Road, Bangor, Gwynedd LL57 2RJ UK

**Keywords:** Realist synthesis, Mixed-methods systematic review, Adolescent, Teen, Pregnancy

## Abstract

**Background:**

Previous research has demonstrated emotional, psychological and educational harm to young mothers following unintended conceptions. The UK has one of the highest rates of pregnancies in adolescence in Western Europe with a high proportion of these being repeat pregnancies, making it a topic of interest for public health policy makers, and health and social care practitioners. As part of a wider mixed-methods systematic review, realist principles were applied to synthesise evidence about interventions aiming to reduce repeat pregnancies in adolescence.

**Methods:**

A multi-streamed, mixed-methods systematic review was conducted searching 11 major electronic databases and 9 additional databases from 1995 onwards, using key terms such as pregnancy, teen or adolescent. The principles of realist synthesis were applied to all included literature to uncover theories about what works, for whom, how and in what context. Initial theory areas were developed through evidence scoping, group discussion by the authors and stakeholder engagement to uncover context + mechanism = outcome (CMO) configurations and related narratives.

**Results:**

The searches identified 8,664 documents initially, and 403 in repeat searches, filtering to 81 included studies, including qualitative studies, randomised controlled trials, quantitative studies and grey literature. Three CMO configurations were developed. The individual experiences of young mothers’ triggered self-efficacy, notions of perceived risks, susceptibility and benefits of pregnancy, resulting in the adolescent taking control of their fertility and sexual encounters. The choice between motherhood and other goals triggered notions of motivations, resulting in the adolescent managing their expectations of motherhood and controlling their fertility and sexual encounters. Barriers and facilitators to accessing services triggered notions of connectedness and self-determination; resulting in interventions that are tailored so they are relevant to young persons, and improve access to services and engagement with the issue of pregnancy in adolescence.

**Conclusions:**

Pregnancy in adolescence is a complex issue with many factors to consider. The conceptual platform described here could help guide policy makers and professionals towards a number of areas that need to be attended to in order to increase the likelihood of an intervention working to prevent rapid repeat pregnancy in adolescence.

**Trial Registration:**

PROSPERO CRD42012003168

**Electronic supplementary material:**

The online version of this article (doi:10.1186/s12884-016-1066-x) contains supplementary material, which is available to authorized users.

## Background

Previous research has highlighted the emotional, psychological and educational harm to young mothers following conceptions in adolescence [1–5]. Across the world, 16 million children annually are born to adolescent mothers, defined as mothers aged between 15 and 19 years old [6]. Though the rate of conceptions in adolescence in the UK in 2012 has fallen by more than a quarter (26.8 %) since 2004, the UK still has one of the highest birth rates among 15–18 years old in Europe [7]. Pregnancy in adolescence is associated with poor educational achievement, poor physical and mental health, poverty and social isolation [8]. As socio-economic disadvantage is seen as both a cause and consequence of pregnancy in adolescence [8], the UK government has included conceptions in women under 18 years as one of its three sexual health indicators, and it is one of the national measures of progress on child poverty [7].

There are a range of theoretical models that underpin interventions designed to reduce first pregnancy in adolescence, such as social cognitive ecological theory [9], developmental theory [10], and psychosocial models (e.g. Health Belief Model [11]). Previous research appeared to show that interventions focusing upon sex education and personal development through skills training may be effective at reducing first pregnancies in adolescence [12–14]. In order to explore what strategies and interventions may be effective for repeat pregnancies in adolescence, a mixed-method systematic review was conducted [15, 16]. The mixed-method review was undertaken as part of a themed call from the NIHR HTA, entitled ‘interventions for preventing repeat unintended pregnancies among adolescents’.

One approach employed within this review was realist synthesis, to provide a theory driven approach to discover what works, for whom, how and under what circumstances for repeat teenage pregnancy. The realist synthesis aimed to identify the mechanisms or programme theories by which the authors postulate the intervention works or explain why the intervention did not work.

### Realist synthesis

A realist review adopts a theory-driven approach to evidence synthesis, which is underpinned by a realist philosophy of science and causality to investigate why and how interventions might work and in what contexts [17, 18]. The aim of this type of review is to then articulate underlying programme theories, interrogating existing evidence to find out if these theories are confirmed or contradicted [17]. In realism, theory is constructed and framed in terms of a proposition or hypothesis about how, and why interventions work (or not). Realist review methodologies are becoming more prominent, and have been applied to school feeding programmes [19], the use of health workers for delivering child health intervention in low and middle income countries [20], and to understand the mechanisms of inter-professional teamwork in health and social care [21].

Realist methodologies were employed as part of a larger mixed-methods review to answer what works, for whom, how and under what circumstances to reduce repeat pregnancies in adolescence. To our knowledge this is the first application of this approach to address the issue of pregnancy in adolescence.

## Methods

### Search strategy

Following scoping searches and piloting of search terms, we searched 11 major electronic databases and 9 additional databases in health and social sciences from 1995 onwards during May and July 2013, and updated the search in June 2014 (see Whitaker et al., 2016 [15] for list of databases and search terms used). Databases included Medline, EMBASE, Cochrane Library, CINAHL, ASSIA, NHS Economic Evaluation Database, EconLit and grey literature (e.g. OpenGrey, Scopus, RePEc and Google). No language restrictions were imposed during the searches; however, grey literature searches were limited to UK only, to enhance applicability of results to UK public health agencies. The search strategy included index terms and text synonyms of key terms such as pregnancy, teen or adolescent.

### Inclusion/exclusion criteria

The realist synthesis utilised all included evidence from the wider mixed-methods review. Using a similar strategy to a previous Cochrane review undertaken in this area [13]; studies were included in the mixed-methods review if the population of interest comprised young women 19 years or younger at the time of conception, who had at least one unintended pregnancy, whether outcome was termination, miscarriage or delivery. Studies were also included if at least 75 % of the target population included young mothers in the target age group stated above. Studies were included if they reported any intervention designed to reduce or delay repeat pregnancies, delivered in any setting (e.g. community, educational). Studies that reported risk factors of repeat pregnancies and barriers to and facilitators of implementation and take up of intervention were also included. Full inclusion criteria and a list of excluded studies, with their reason for exclusion can be found at Whitaker et al. [15].

### Quality appraisal

The studies included in the mixed-methods review and the realist synthesis were subject to quality appraisal using the Cochrane Collaboration’s Risk of Bias [22] for trials; Mixed Methods Appraisal Tool (MMAT) [23] for qualitative studies and surveys, and Drummond Checklist for a sound economic evaluation [24]. The strength of the evidence provided by the randomised trials was appraised using GRADE [25] and evidence provided by the qualitative synthesis using CERQual [26]. In accordance with the principles of realist synthesis, included studies were further quality appraised using the principles of relevance and rigor [18]. Relevance questions whether the study can contribute to theory testing or building. Whilst, rigor questions whether the original research has sufficient weight to make a methodologically credible contribution to the testing of a particular intervention theory. Rycroft-Malone et al [27] applied a similar approach regarding the principles of relevance and rigor in their realist review exploring skills and care standards in the support workforce for older people, which we used as an exemplar and applied to our review.

### Involving stakeholders

During the course of the mixed-methods review, three events were held with key stakeholders. Two included representatives from public health, primary care, sexual health, obstetrics, midwifery, whilst the third included adolescent mothers. These meetings were held to discuss methodology, present findings and receive feedback. Realist review methods and findings were also presented and discussed at all three stakeholder meetings.

### Theory development

Initial theories were developed by exploring the evidence and group discussion between the authors. This involved a high-level theming process managed through deliberative processes, which were informed by the original brief, review questions, emerging evidence and other policy documents. This process resulted in a list of questions of interest that covered a number of different levels; for example, the individual level, the intervention level and the team who deliver the intervention.

These questions were further mapped in an iterative manner into key theory areas and a mind map was developed to accompany these areas with key words illustrating each theory area. Meanwhile, the mind map and theory areas were shared with stakeholders as part of consultation meetings for the full mixed-methods review. Following these meetings, the theory areas and mind map were amended to reflect comments received from the stakeholders (see Additional file 1). The authors met regularly expanding and refining theories until they reached a consensus of the final list of theories. This final list was then applied to all the included studies in the review. The final theory areas were: Connectedness (e.g. social support, peers, partners); Structure (e.g. Individual versus group-led interventions); Tailoring of the setting or environment on an individual level (e.g. personalised, linked to other services) or on an intervention level (e.g. community versus educational setting); Motivation - Reasons for getting pregnant; Other Goals/Aspirations (e.g. education or vocation goals such as going to university or planning a particular career path); and Perceptions/ideas of parental responsibility (e.g. motherhood seen as a desired role).

### Data extraction

Bespoke data extraction forms were created in Microsoft Access where a space was provided for the reviewers to extract evidence pertaining to a particular theory area for all included literature in the review. The reviewers searched the included studies for explicit theories, for example, psychological models (e.g. Health Belief Model [11]). The reviewers also searched for implicit theories, for example, when authors postulated causality or factors that increased or decreased the likelihood of repeat adolescent pregnancy. Data extraction was conducted independently by two reviewers, and disagreements resolved by a third reviewer if necessary. The principles of realist synthesis were applied to all included literature to uncover theories about what works, for whom, how and in what context.

An *aide memoire* (crib sheet) was developed for supporting data extraction pertinent to the realist synthesis of the review. For each theory area, explanations, meanings and key words were listed. Any new information found by the reviewers that did not fit the already established theory areas were highlighted via e-mail, with the new heading name, an explanation of what this theory area contained and key words. The crib sheet was then updated accordingly, and throughout the data extraction process. Once the reviewers had completed their first and second extraction of assigned included papers, they shared their summaries of the realist evidence. Tables were compiled showing the author, year, verbatim data extraction, the reviewer’s own personal commentary on the data, and how it confirmed, refined or contradicted the theory as per realist methodology [28]. The reviewers also provided their interpretation of explicit theories and author postulations (implicit theories) in the commentary section for the data extraction forms. The first and second author then gathered the evidence for each theory area creating a narrative of the theory.

### Data synthesis

The evidence gathered for each theory area developed into a narrative, with common mechanisms and contexts visible to the first and second authors. Further scrutiny and analysis of this emerging narrative, across each theory area, resulted in the emergence of a number of key mechanisms that seemed to be potentially important for increasing the likelihood of interventions working. We also identified a number of different contexts in which these mechanisms may (or may not) be triggered. These narratives were then moulded into CMO configurations as the story naturally developed as to how certain contexts triggered certain mechanisms to arrive at a particular outcome. The results are presented according to RAMESES publication standards for realist synthesis [18] (see Additional file 2).

## Results

The searches identified 8,664 documents initially, and 403 in repeat searches, filtering to 10 Randomised Controlled Trials (RCTs), four quasi RCTs, 10 qualitative and 53 other quantitative studies published from 1996–2013. Four documents found in the grey literature searches were also included in the realist synthesis. For a detailed flow diagram of identified studies, screening, eligibility and inclusion please see Whitaker et al [15]. Three CMO configurations were developed. Table 1 summarises the key information from each CMO configuration. Following Table 1 each CMO configuration is presented in turn.

**Table 1:** Summary of the three CMO configurations

Context	+	Mechanism	=	Outcome
**Individual Experiences** Age, Culture, Past Experiences Family, peer and community influences, Personal circumstances and Past/current relationships		Taking control triggered through self-efficacy & perceived risks, susceptibility and benefits of pregnancy		If the adolescent views pregnancy as a likely negative outcome, with severe consequences and little benefit, they will take control of perceived barriers overcoming them to protect themselves against pregnancy.
**Barriers and Facilitators** Developmental stage and age, Knowledge of services, contraception availability Contraception preferences, Issues of access, Incentives, crèche facilities, and transport		Tailoring triggered through connectedness, support and self-determination		Tailoring interventions so they are relevant to a young person may result in a greater potential for connectedness with the intervention and the issue of teenage pregnancy itself, providing a notion of support and triggering self-determination. Feeling connected and supported can help an adolescent feel their life choices are being encouraged. A supportive professional delivering the intervention, the group itself or family members can help to verbalise and confirm a mother’s skills, and help them to develop strategies and plans to change their attitudes and behaviour.
**Motherhood versus Other Goals** Thoughts and feelings about motherhood, Emphasis placed on motherhood, How do adolescents plan or view the future, Engagement with school or a vocation		Motivations triggered by self-esteem, self-efficacy & empowerment		Motivations (conscious or unconscious) could lead the adolescent to manage their expectations of motherhood and take control of sexual encounters resulting in consistent use of contraception, if they feel there are other goals or opportunities available outside of motherhood to achieve success.

### Conceptualising the issue of pregnancy in adolescence

In the evidence base, there was a polarisation with regards to the epistemology of interventions to prevent or delay subsequent teenage pregnancy. Authors of quantitative studies viewed pregnancy in adolescence from the perspective of irresponsible, problem behaviour. Conversely, authors of qualitative studies viewed teenage pregnancy from the perspective of the adolescents or health-care professionals who work closely with young mothers. In the synthesis below, we applied realist principles, ascribing to multiple perspectives (from the adolescents themselves to policy makers) and were guided by the evidence, rather than having an *a priori* stance on the issue.

### CMO 1: Taking control of the issue of adolescent pregnancy

There is a broader context surrounding adolescents that needs cognisance. Adolescents will have their own experiences for example: past pregnancies [29], perhaps a history of loss or miscarriage [30] and even incidences of partner violence [31]. Past and current relationships can increase the chances of an adolescent having a rapid repeat pregnancy. Partners may influence childbearing, particularly if they are married, co-habiting or in an on-going relationship with the adolescent [32–34]. Knowledge about sexual health and services, including contraceptive preferences [35] and choice of contraceptive methods [36] can also play a part in the likelihood of an adolescent experiencing a rapid repeat pregnancy. The adolescent will also have their own thoughts and feelings about motherhood, and their peers, family and community could influence this [9, 37–39]. Adolescents may be exposed to friends and family that place a high emphasis on motherhood and parenting [9, 37, 39]. This emphasis may align with the adolescent’s own thoughts and feelings or it may not.

Under this context, external influences, including knowledge and engagement with pregnancy assimilates into the adolescent’s own thinking. Depending upon whether they feel at a risk of pregnancy and whether becoming pregnant at this stage is their lives would be a positive or negative consequence, can trigger the mechanism of taking control. This could be taking control of their own fertility by using hormonal contraceptives, sexual encounters by choosing to engage in them or not, and whether to insist on the use of prophylactics such as condoms. Mbambo [40] drew on the Health Belief Model [11] to provide a construct of how an adolescent may internalise their susceptibility of getting pregnant again, the perceived severity of getting pregnant (e.g. breakdown in family relationships), and perceived benefits of not getting pregnant again (e.g. completing schooling). These susceptibilities, severities and benefits are appraised against variables affecting the initiation of actions (perceived barriers). For example, problems accessing contraceptives, limited knowledge and lack of supportive individuals to discuss sex and relationships. This in turn may lead the adolescent to either access or ignore services, taking or avoiding precautions.

If the adolescent views pregnancy as a likely negative outcome, with severe consequences and little benefit, they will take control of perceived barriers overcoming them to protect themselves against pregnancy [35, 36, 40]. Conversely, an adolescent that views pregnancy as a likely positive outcome, with minor consequences and large benefit, will take control of sexual encounters, and perhaps avoid precautions [35, 40].

### Actionable implications

As part of the service, medical professionals could provide contraceptive counselling as well as the actual contraception in order to capture issues outside of the medical paradigm [35]. This would provide a more holistic approach exploring the reasoning and context behind contraception and service choices, especially as these could change over time. There could also be opportunistic timing at the initial pregnancy that could help prevent a subsequent pregnancy. This widening of approach also needs to consider the emotional and psychological barriers to contraceptive use [41]. Designing and delivering interventions that promote self-efficacy and empowerment, and understand perceived risks, susceptibility and benefits of pregnancy from the adolescent’s perspective could provide them with the opportunity to take control of sexual encounters, contraception and decisions about pregnancy (Table 2).

**Table 2:** Example extracts from the literature and stakeholder events used to develop CMO 1

*CMO 1 - Individual experiences of young mothers trigger self-efficacy, perceived risks, susceptibility and benefits of pregnancy, resulting in the adolescent taking control of their fertility and sexual encounters.*
Paukku et al [29] “Adolescents’ contraceptive practice differed remarkably by pregnancy history.”
Barnet et al [32] “Similar to findings of previous studies, our findings show an increased subsequent pregnancy risk for an adolescent mother who reports a romantic relationship with the baby’s father.”
Black et al [9] “Finally in some communities, rapid second births among adolescent mothers may be valued and regarded as desirable, thereby undermining many intervention programs…”
Herrman [39] “In low-income communities there may be contextual rewards for bearing children, where the norms might not discourage adolescent childbearing.”
Bull & Hogue [37] “....in school all the girls either got babies or is expecting…it’s got to the point where it’s normal in a group of girls; if you haven’t been pregnant, then you don’t fit in”
Clarke [41] “…a purely mechanical approach to contraceptive provision is very unlikely to work for many young people…Therefore, contraceptive providers…need to widen their approach to ensure that there are opportunities for the many emotional and psychological barriers to contraceptive use…”
Stakeholders involved in the research stated “If their first pregnancy resulted in termination, still birth or the child was taken into care, they want to replace a baby…these young women do not receive any bereavement counselling.”

### CMO 2: The adolescent’s motivation to engage with the issue of adolescent pregnancy

Each person will have different reasons for becoming pregnant. Adolescents are influenced by community norms [9, 42, 43]; social context [38, 39]; peer influence [9, 32–34, 37, 39] and experiences [29–31], which all combine to create a complex contextual mix. These contexts can trigger the mechanism of motivation (consciously or unconsciously) to become pregnant or protect against pregnancy.

In the literature the motivation(s) to engage with the issue of pregnancy, whether conscious, unconscious, positive or negative, were contingent upon the issue of how young women plan or view the future. Evidence suggests that adolescents perceive a dichotomous choice between motherhood [9, 30, 33, 39] and other goals and aspirations [9, 34]. In the absence of other goals and aspirations, some adolescents may look to motherhood as a conscious choice. Low educational attainment and disengagement with school may lead adolescents to believe childbearing provides them with an opportunity to experience success, gain autonomy and self-esteem that they cannot gain through academic or vocational routes [9, 34]. Stakeholders consulted as part of the evidence gathering raised concerns that giving young mother praise during pregnancy and the early years may be the only time an adolescent has felt she is good at something. If this is the only context that a mother receives praise and she does not have any opportunities outside of motherhood to feel praise; then pregnancy is being positively reinforced by those around her, and could lead to further pregnancies in order to maintain the praise.

Based upon the adolescent’s experiences and past an adolescent may become motivated, whether consciously or unconsciously to become pregnant. These motivations are particularly relevant at a time in the adolescent’s life when they are developing independence, and preparing for vocational roles or further academic study as they approach their final mandatory schooling years [9]. Motherhood may provide security and stability in their lives otherwise characterised by instability, detachment and low expectations. Repeat pregnancies may provide a route for continued stability in the form of contact with healthcare professionals and other mothers. If an adolescent feels their options are limited, they may feel that motherhood offers the only way to experience success in life [30, 33, 39]. They may also believe that their choices do not impact greatly upon their life, leading to impulsive decisions, without thinking through the consequences [39, 44].

Motivations could lead the adolescent to believe that motherhood provides the only opportunity to experience autonomy and success in life, resulting in them taking control of the situation, seeking out partners and opportunities to engage in sexual activity [9, 30, 33, 39]. Conversely, a belief that other goals or opportunities are available to achieve success outside of motherhood could lead the adolescent to manage their expectations of motherhood, and take control of sexual encounters resulting in consistent use of contraception, [9, 34]. The adolescent may reduce their chances of pregnancy if they feel motherhood may reduce the likelihood of them achieving these external goals [34].

### Actionable implications

Understanding and working with the different motivations underpinning decisions about pregnancy could facilitate the adolescent taking [more] control. Paying attention to self-esteem, self-efficacy (confidence in one’s ability to take action) and empowerment were all stated as ways that could increase the likelihood of motivating an adolescent to take control; [3, 9, 30, 32, 37, 39, 45, 46], and thus help the adolescent to engage with the issue of pregnancy in adolescence, and [better] manage their expectations of motherhood. An adolescent may expect they could cope with motherhood and seek out motherhood, or they could feel they would not cope and thus avoid motherhood (Table 3).

**Table 3:** Example extracts from the literature and stakeholder events used to develop CMO 2

*CMO 2 – Choice between motherhood and other goals, triggers notions of motivations (conscious or unconscious) could lead the adolescent to manage their expectations of motherhood and take control of sexual encounters.*
Raneri & Wiemann [33] “Nevertheless, medical and social service providers should help young mothers to think about how potential age, financial or relationship imbalances may affect decisions regarding childbearing and other life plans.”
Herrman [39] “when asked about decision making before unprotected sexual activity, the mothers claimed that either they did not weigh the consequences or that the consequences were not powerful enough to alter behaviours.”
Raneri & Wiemann [33] “…Some teenage mothers may be ambivalent about using contraceptives to prevent additional pregnancies. Others feel they have limited educational and occupational options, and that early motherhood is not tragic or even problematic choice.”
Barnet et al [32] “Motivational interviewing is a counselling style that emphasizes an individual’s personal goals and self-efficacy in relation to complex health behaviours…Motivational interviewing aims to highlight discrepancies between current behaviors and personal goals, thereby promoting an intention and optimism for change.”
Carvajal et al [45] “Positive provider communication is associated with pregnancy prevention self-esteem.”
Clarke [30] “…commitment to their maternal identities, provided a buffer against the potential threats to self-esteem.”
Stakeholders involved in the research stated “Teenagers may be getting pregnant as there are no jobs, no prospects. Girls sometimes “drift”, thus they just continue the pregnancies because they do not know there are other options.”
Stakeholders involved in the research stated “…there is a need for increased self-esteem, life skills and empowerment of teenage girls.”
Stakeholders involved in the research stated “Need to empower the girls to make choices, this message needs to be given by all services consistently.”

### CMO 3: Tailoring so interventions work with the adolescent and their circumstances

The issue of adolescent pregnancy involves family, societal, medical and educational components [9, 29, 37–39]. Therefore, given this complex contextual mix, interventions need to address the wide-ranging and complex needs of the young women, not just the issue of pregnancy at an early age [47]. Interventions need to be *situated within the wider context* of the young person, to ensure they are suitable and preferred. Interventions should pay attention to adolescent’s life experiences [29–31, 48], as well as their developmental stage [44], cultural context, age-appropriate impulsive and rational decision-making styles [45] and responses to stress [39]. Interventions that attend to these contexts may be more successful than interventions based upon a purely medical model [41]. Medical models are defined as interventions that focus on providing information and access to contraception only; such as, the suggestion that the most effective way to help adolescent mothers might be to encourage them to use long-acting injectable or implant contraception methods [29, 49]. However, young mothers attending a service user feedback group highlighted experiencing side effects and in particular, the lack of suitability of long acting reversible contraception when it is required to be removed and a new implant/device fitted. The mothers highlighted that they were placed back onto daily oral contraceptives for around 3 weeks before a new implant was fitted. The young mothers stated that daily oral contraceptives did not work for them; they tended to forget to take them, which is why they preferred long acting methods. Some expressed they got pregnant during these weeks, as they did not have methods in place that suited them, and thus they were not used effectively.

Tailoring provides a mechanism to situate the intervention in a young person’s life. Interventions need to be tailored to address practical, emotional and psychological barriers [41, 47], for example, practical issues of access. Adolescent mothers face a difficult course navigating obstacles such as school failure and repeat pregnancy, increasing the risk of school dropout and persistent poverty [47]. Interventions reach these adolescents more effectively when located in accessible sites such as at school or home [47, 50]. Incentives can be used as facilitators to increase intervention uptake and engagement with the issue; for example, providing monetary incentives such as food vouchers [37] or parenting classes and crèche facilities [33, 51]. Stakeholders and young mothers involved in the research also highlighted the need for transport if the intervention is to be delivered outside the home, for example in a community centre. The stakeholders and mothers also highlighted flexibility as an important facilitator. Interventions that provide flexibility allow the adolescents to take or collect their child/ren from school, take account of difficulties in getting children ready to leave the house and absences or lateness due to child sickness. Interventions also need to include an understanding of adolescent sexual behaviour from the point of view of the adolescents themselves. Many interventions are designed based upon adult beliefs of adolescent sexual activity and the consequences of adolescent pregnancy, which lack relevance to the adolescent population the intervention aims to serve. The evidence suggests much of adolescent sexual activity is spontaneous, unplanned, and sometimes involuntary [32, 33, 39].

Interventions should be developed using adolescent principles and perceptions of sex and relationships and the potential consequences of pregnancy during adolescence [44]. For example, understanding the spontaneity of sexual encounters, the influence of peers and feelings of love and commitment with a partner.

Tailoring interventions so they are relevant to a young person results in a greater potential for connectedness with the intervention and the issue of adolescent pregnancy itself, providing a notion of support and triggering self-determination. Feeling connected and supported helps an adolescent to believe their life choices are being encouraged [9, 52]. A supportive professional delivering the intervention, the group itself or family members can help verbalise and confirm a mother’s skill [53], and helps them to develop strategies and plans to change their attitudes and behaviour [54]. Feeling connected and supported as part of a group of mothers to whom they can relate, inspires mothers to achieve, and not be limited because of their current situation [52]. Young mothers attending a service user feedback group also stated that being part of a group allowed them to hear others’ feelings and opinions and let them know that they were “not alone”.

### Actionable implications

Policy makers and intervention developers need to tailor interventions within a broader context, with reference to multiple influences and the roles that an adolescent mother has to play including student, employee, friend and daughter. Interventions should be developed using adolescent perceptions of sex and relationships so they are relevant to the adolescent. Tailoring interventions can increase the adolescent’s level of connectedness with the issue of adolescent pregnancy. Being connected to the issue or a particular intervention provides a notion of support, triggering self-determination and enabling the adolescent to steer their life-course in their preferred direction (Table 4).

**Table 4:** Example extracts from the literature and stakeholder events used to develop CMO 3

*CMO 3 - Barriers and facilitators to accessing services, trigger notions of connectedness, support and self-determination; resulting in the tailoring of interventions so they are relevant to young persons and improve access to services and engagement with the issue of pregnancy in adolescence.*
Clarke [41] “…a purely mechanical approach to contraceptive provision is very unlikely to work for many young people…Therefore, contraceptive providers…need to widen their approach to ensure that there are opportunities for the many emotional and psychological to contraceptive use…”
Haamid & Wiemann [44] “There was a lack of intention associated with repeat pregnancy…The adult who expects young people to engage in premeditated sex after deliberating the costs and rewards of an unintended pregnancy is not looking at sex and pregnancy from an adolescent viewpoint…interventions must be created that take the nature of this sexual activity into account.”
Key et al [47] “Adolescent mothers face a difficult course navigating obstacles such as school failure and repeat pregnancy.”
Stakeholders involved in the research stated “Schemes that involve home visits are likely to be more successful than involving people getting to clinics using public transport.”
Stakeholders involved in the research stated “Childcare to support young mothers going back into education should be more supported and facilitated”.
Stakeholders involved in the research stated “Interventions need to be tailored to the individual according to circumstances at the time – girls complain that they are not listened to by professionals”.
Stakeholders involved in the research stated “We really need to find out what they really need and want and understand what they are asking for – there is too much generalisation, and perceptions of what girls want are not accurate”.
Young mothers in a service user feedback group stated they would like services/interventions to have; “Positive discussion about contraception choices, teen centred groups and flexible services that allow for travel issues, difficulties in getting children ready to leave the house and absences due to child sickness.”
Young mothers in a service user feedback group stated “In a group, we can see everyone, hear everyone’s opinion and if I feel something I am not alone.”

## Discussion

### Main findings

Pregnancy in adolescence is a complex issue, with many factors to consider. The adolescents themselves need to be engaged in the issue, they need to know that they are being listened to and their choices are being reinforced. Practitioners need to be aware of potential barriers and facilitators, such as suitability and preferences of methods (e.g. long acting reversible contraceptives versus daily oral contraceptives). The CMOs we developed highlight adolescents face multiple influences, choices between motherhood and other goals and differing levels of knowledge, services and access. How an adolescent internalises these influences and their environment can trigger feelings of *taking control*. In the literature, adolescents either view pregnancy as a severe consequence with little benefit and protect themselves against pregnancies, or conversely, the adolescent can view pregnancy as something to be desired with personal benefits, and take control to become a mother. How an adolescent internalises the dichotomous choice between motherhood and other goals such as further education could lead them to be *motivated* (consciously or unconsciously) to engage with the issue of pregnancy in adolescence. Engagement with the issue could lead to adolescents exploring their contraceptive options, using them consistently, managing their expectations of motherhood, and taking control of sexual encounters if they do not yet want to become pregnant. The mechanism of tailoring can provide lessons for service providers. It is important to empower adolescents, by making them feel they are being listened to, and providing a supportive environment so they feel others are reinforcing their choices. There are also wider issues to consider such as clinic opening hours and their location. Adolescents attending school or college need to access services outside of the school day. If clinics or GP surgeries are not open outside these hours, then this restricts their access and creates a barrier. Additionally, adolescents are reliant upon public transport or family members to access services if they are not within walking distance. For some, there is an open discourse within the family about sex and relationships, which may allow the adolescent feel comfortable to ask for assistance in getting to a clinic or GP surgery. However, for others they may not feel comfortable in discussing such matters with their families providing obstacles and barriers to access, which they must overcome alone. Service providers should be aware of barriers (e.g. issues of access) and provide solutions, such as longer opening hours, consideration of the location of services and use of schools and school nurses to support adolescents. The main findings of the review should be subject to future research, before being implemented in interventions or developed into policy.

### Quality of the evidence base

The standard for reporting in the evidence base was mixed. However, most studies were considered to be of good quality. Using the principles of relevance and rigor, the included evidence confirmed middle range theories, helped to refine our thinking and made a credible contribution to theory development.

### Cultural context of evidence

The review found limited UK evidence; we used UK grey literature and stakeholder consultation to inform the realist review to enhance applicability of results to UK public health agencies. There is a need for recognition of wider cultural perceptions of motherhood in adolescence. For some cultures, pregnancy in adolescence is viewed as the social norm and the role of mother is considered a desirable one. In other cultures, there is stigma around pregnancy in adolescent and judgement of young mothers. In the realist synthesis, we theorise it is how an adolescent internalises these external factors and influences with their own thinking to derive at their own perceptions of motherhood, pregnancy, sex and relationships, to trigger mechanisms such as motivation and control. There are also issues of access and funding for health care services that offer contraception and family planning advice in different contexts. The realist synthesis highlights the mechanism of tailoring by policy makers and service providers to reduce barriers and increase facilitators to access of services and uptake of interventions.

### Strengths

We applied realist principles as part of a wider mixed-methods review, which included a varied range of literature from RCTs to qualitative studies and grey literature giving our analysis a rich source of evidence. Unlike previous studies [19] realist methods were applied whilst undertaking the wider review. Therefore, rather than being directed firstly by the systematic review findings and then exploring the literature using the realist approach, realist methodologies drove the process from the start. We also involved stakeholders throughout the process and discussed theory development and our interpretation of the evidence base, which further strengthen the review, and has been previously utilised by others [55, 56].

### Limitations

Though RCTs are considered by most to be the gold standard of evidence, they are usually disseminated with limited description or explanation as to why an intervention worked. Therefore, it was left to the reviewers’ interpretation of the evidence to determine whether a theory was explicit or implicit. The interpretation of evidence, though a key component of realist principles, has implications for the replications of findings from the review, as others may have interpreted the evidence differently. To minimise this limitation as much as possible, we involved stakeholders from public health, primary care, sexual health, obstetrics, midwifery and adolescent mothers throughout the review.

## Conclusions

Adolescents face multiple influences, choices between motherhood and other goals and differing levels of knowledge, services and access. How an adolescent internalises these influences and their environment can trigger *taking control*, *motivations* and *tailoring*, leading to outcomes that can either reduce or increase repeat pregnancy in adolescence. The conceptual framework outlined in this paper can help guide policy makers and professionals towards a number of areas that need to be attended to in order to increase the likelihood of an intervention working to prevent rapid repeat adolescent pregnancy.

## Additional files

Additional file 1: Mind Map of key areas highlighted by scoping of the literature, with additional points raised by stakeholders highlighted. (DOCX 193 kb) Additional file 2: Completed RAMESES Checklist for the realist synthesis presented. (DOCX 15 kb)

## Abbreviations

CMO: Context + Mechanism = Outcome
MMAT: Mixed methods appraisal tool
RCTs: Randomised controlled trials
