# Supplementary material for: Reducing repeat pregnancies in adolescence: applying realist principles as part of a mixed-methods systematic review to explore what works, for whom, how and under what circumstances
Source: BMC Pregnancy Childbirth. 2016 Sep 20;16:271. doi: 10.1186/s12884-016-1066-x (PMC5029024; doi:10.1186/s12884-016-1066-x)

Additional File 1. Mind Map of key areas highlighted by scoping of the literature, with additional points raised by stakeholders highlighted.


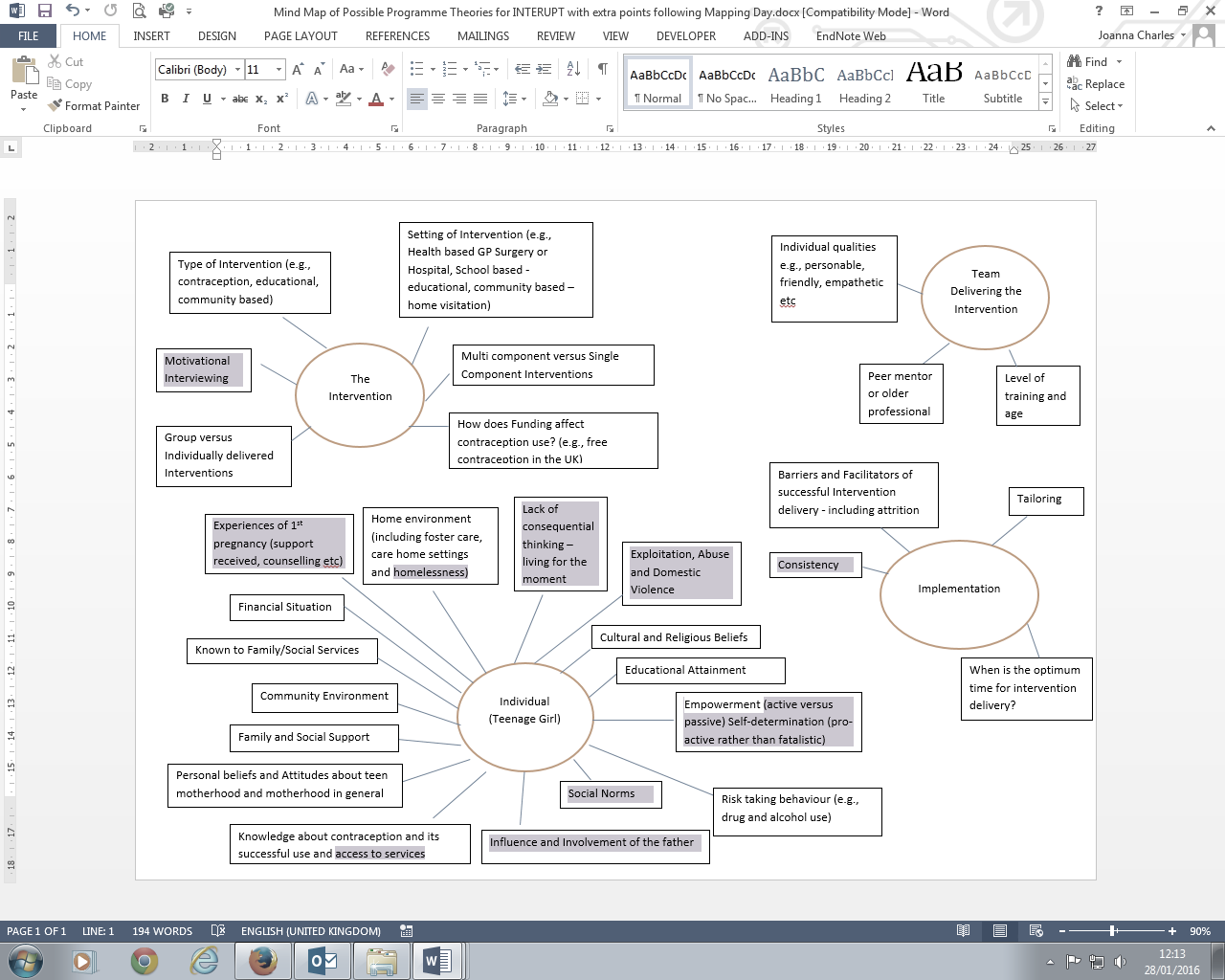

Supplement: Additional file 1: — Mind Map of key areas highlighted by scoping of the literature, with additional points raised by stakeholders highlighted. (DOCX 193 kb) [file 12884_2016_1066_MOESM1_ESM.docx]
